# Research circles as a method for implementing new services in the public health and welfare system

**DOI:** 10.1080/17482631.2024.2366087

**Published:** 2024-06-11

**Authors:** Birthe Møgster, Ottar Ness, Monika Alvestad Reime

**Affiliations:** a Department of Welfare and Participation, Western University of Applied Sciences, Bergen, Norway; b Department of Education and Lifelong Learning, Norwegian University of Science and Technology, Trondheim, Norway

**Keywords:** Research circles, co-creation, service interventions, public values, action research, innovation capacity

## Abstract

**Background:**

Co-creation has become a guiding principle in public service innovation, but more knowledge is still needed on overcoming barriers and increasing the effectiveness of co-creation processes. This study explores the research circle method as a concrete methodology for co-creation, and its application within two cases involving the implementation of new services for drug death-bereaved persons in Norway based on new research-based knowledge.

**Method:**

The study followed an action research design. The field notes and audio recordings were analysed using reflexive thematic analysis.

**Results:**

The analysis identified two key dimensions experienced as important for the implementation of the new services when research circles were used as a method for co-creation: 1) the inclusion of participants from different contexts and 2) support structures for service interventions.

**Discussion:**

Research circles are discussed as an important support structure for promoting public value co-creation that can contribute to increasing stakeholders’ capacity for implementing services in the public system, especially when the focus is on the perspectives and interests of stakeholders, such as practitioners and management in public health and welfare services. However, the discussion also points to barriers relating to the co-creation process that need to be considered when planning research circle-based interventions.

## Background

In recent years, co-creation has gained increased attention as a guiding principle for public sector innovations that contribute to solving the sector’s complex problems (Hartley et al., 2013; Osborne, 2021; Torfing et al., 2019). However, identifying key dimensions for the co-creation process and possible outcomes is essential for the effective implementation of new services in the public sector (Hartley et al., 2013; Osborne & Brown, 2011; Torfing et al., 2019). In this article we present and discuss research circles as a method for co-creation that has been used for the development and implementation of new services (service interventions) to support bereaved individuals after drug-related deaths. These fatalities have become a major public health problem worldwide (Centers for Disease Control and Prevention, 2024; European Monitoring Centre for Drugs and Drug Addiction, 2023), and the health consequences for the bereaved persons can be severe (Bottomley et al., 2022; Christiansen et al., 2020; Djelantik et al., 2020; Titlestad & Dyregrov, 2022). Research has documented a need for developing targeted and needs-based services to prevent the possible harmful consequences following such bereavements (Fjær & Dyregrov, 2021; Kalsås et al., 2023).

Co-creation and innovation in the public sector involve a collaborative effort between public and private actors to achieve shared aspirations and an exchange of resources, and require an extended level of participation (Osborne, 2021; Torfing et al., 2019). Co-creation aims to bring together resources and perspectives from various stakeholders, including service users, to create more effective and user-centred services (Torfing et al., 2019), encourage innovation (Torfing et al., 2021), and together create values in “the nexus of interaction” (Osborne, 2018, p. 225).

Several researchers have highlighted existing barriers to public sector co-creation and innovation (Acar et al., 2023; Cinar et al., 2019; Hartley et al., 2013; Osborne & Brown, 2011; Torfing et al., 2019), such as a lack of outcomes (De Vries et al., 2016) or objectives (Voorberg et al., 2015), or co-destruction when co-creation leads to the failure of public value (Acar et al., 2023; Osborne, 2021).

### Co-creation and its outcomes in the health and welfare system

The health and welfare system is not exempt from the need to transform and improve services, and co-creation has emerged as a potential solution to address the various challenges faced (Acar et al., 2023; McMullin, 2023; Torfing et al., 2023). The focus on co-creation in recent years has also influenced innovations in the health and welfare services and the creation of public values (Acar et al., 2023; Palumbo, 2016). Bozeman (2007) defines public values as normative consensus about the key values in society. Furthermore, Acar et al. (2023) suggest that co-creation outcomes can be translated into public values and classified into three categories: service, relationship, and democratic qualities. Service community values contribute, among other aspects, to efficiency and service quality improvement. Those related to relationships concern, among other things, improving citizens’ learning environments, power relations, and working conditions. Democratic qualities emphasize the promotion of democracy in co-creation processes, including empowerment, inclusion, and participation (Acar et al., 2023).

The demands for services to be based on research-based knowledge have become increasingly important, leading to new challenges regarding its inclusion (Gulbrandsen et al., 2021). The non-integration of research-based knowledge into health and welfare services has led to a research-practice gap (Gulbrandsen et al., 2021). Studies have shown that implementing research-based knowledge into health services takes many years and pointed to several difficulties in this regard (Gabbay & le May, 2023; Graham et al., 2006; Khan et al., 2021; Morris et al., 2011; Straus et al., 2013). These include problems relating to the integration of professional perspectives anchored within a bio-medical model with lay actors’ knowledge derived from a democratic and emancipatory tradition (McMullin, 2023; Palumbo, 2016; Torfing et al., 2023). Professionals also need to change their roles from acting as providers of services to managing collaborative service provision processes (Steen et al., 2018). Thus, to solve present and future issues, there is a need to explore how to apply research-based knowledge through innovative designs (Gulbrandsen et al., 2021).

Osborne (2021) introduced the framework of the “co-creation of value,” emphasizing that value creation occurs in ecosystems, in the interconnection between the micro- (stakeholders, service providers, and users), meso- (service delivery systems and governance of these systems), and macro- (institutional) levels. The framework Osborne (2021) developed requires a shift to view the value creation process as occurring in internal value chains in the ecosystem. Both the individual (e.g., achieving new skills and competence) and societal (e.g., creating active users in co-creation processes) creation of value occurs during co-creational processes.

The essential factors for an organization’s ability to co-create health and welfare services require strengthening its capacity for innovation. Meijer (2019, p. 617) defined this as “the capacity to develop and realize new ideas for societal problems.” This involves facilitating co-creational skills and supportive systems for innovation. Challenges in building innovation capacity in the public sector include identifying needs, opportunities, or challenges framed as dynamic capabilities, developing and scaling innovations as complementary capabilities, and promoting employees’ commitment as collective engagement, with all three functioning as vital components in successful co-creation (Meyer et al., 2023).

### Research circles as a method for co-creation

In this paper, we explore research circles as a method for co-creation, in which multiple stakeholders contribute to developing and implementing new services for bereaved persons after drug-related deaths. Research circles derive from the *study circles* concept and have a long history in the Swedish educational system as a collaborative participation model based on deliberate and empowering structures between researchers and practitioners (Holmstrand et al., 2008; Persson, 2016). As such, research circles aim to build environments for innovative knowledge creation and sharing where the participants gain increased knowledge and understanding of a chosen topic. Research circles usually consist of three phases: 1) identifying service challenges and gaining relevant knowledge about a topic, 2) developing and testing out measures, and 3) evaluating (Persson, 2016).

In recent years, research circles have gained increased attention for developing service interventions in health and welfare systems in the Nordic countries. They have been used for various purposes, such as local development (Follevåg & Seim, 2021) and increasing user participation in different contexts and among groups of vulnerable citizens (Chalachanová et al., 2023; Follevåg & Seim, 2021; Follevåg et al., 2023), and to implement research-based knowledge (Löfqvist et al., 2019; Møgster et al., 2023). Research circles that include service users as stakeholders all demonstrate an increase in user participation and highlight that participants’ distinct knowledge and experiences bring diverse perspectives into the research circle (Chalachanová et al., 2023; Follevåg & Seim, 2021; Follevåg et al., 2023; Löfqvist et al., 2019; Møgster et al., 2023; Witsø et al., 2022). Löfqvist et al. (2019)

Various works have identified research circles promising to facilitate empowerment and participation among vulnerable citizens. However, these have mainly focused on exploring the process of the research circle and the individual effects of participation rather than how they contribute to concrete service interventions. This study aims to expand knowledge about research circles as a method for co-creating service interventions by exploring the implementation phase of a research circle aimed at facilitating research-based interventions in bereavement care. The research question is: *what key dimensions have been experienced as important for the implementation of new services in the public health and welfare system when research circles are used as a method for co-creation?*

The article proceeds as follows. First, the methods, including a description of the two service interventions, are outlined. The analysis and generation of codes and themes are then described. The findings are discussed in light of the presented theoretical approaches to co-creation. Finally, conclusions and suggested implications for future research are presented.

## Methods

In this action research study, we designed and implemented new services through a co-creation process. Action research involves the development of knowledge, theory, and practice in democratic, participatory, and action-oriented research approaches and implies co-creation using language, learning, and social and practical changes (Bradbury, 2015; Hersted et al., 2020; Rauch et al., 2014). This action research study is positioned within a social constructionist epistemological stance that views knowledge as co-created (Gergen, 2022).

As an action researcher, the first author (BM) participated as a member and co-facilitator of the research circle and as a regular member in the two project groups working with the two service interventions. Additionally, the first author supported the research circle participants in implementing the new services. The last author (MAR) took part in the research circle meetings as the facilitator of the research circle. The first and last authors are members of the research project and were able to provide the service interventions with direct access to the new research-based knowledge. The second author (ON) assisted in the planning and analysis, and all the authors collaborated in writing up the study.

### Study context—the research circle

The present paper explores the action phase (second phase) of a research circle project derived from a large Norwegian study on drug-related death and bereavement (the END project). In the first phase of the research circle activity, the participants worked with the translation of research-based knowledge and planned their service interventions. See Table 1 for an overview of the research circle phases.

**Table I.** Research circle phases and their main content.

Phase	Period	Main content in the research circle phases
Phase 1	August 2021–March 2022	Presentation of relevant knowledge from the END project. Developing ideas for service interventions and planning implementation process
Phase 2	March 2022–November 2022	Implementation of new services (service interventions)
Phase 3	November 2022–March 2023	Evaluations of service interventions and research circle work

The research circle was composed of different participants recruited via the main project’s project group and advisory board: mothers bereaved by drug-related deaths, representing a peer organization (two), practicians from civil societies (from the third sector) (two), public sector providers (three), lecturers (and members of the END project) from the Western Norway University of Applied Sciences (HVL) (two), and researchers from the END project (two), totalling 11 participants.

Several new services were designed and implemented throughout the work in the research circle. In this study, we will explore two of the service interventions in depth: (1) peer support for recently bereaved persons after drug-related deaths and (2) a monthly low-threshold meeting place for bereaved persons after drug-related deaths. The two interventions represent a variety in geography, type of organization, and the participants’ positions.

### The two service interventions: participants and processes

#### Service Intervention 1: Peer Support for Recently Bereaved Persons After Drug-Related Deaths

The two bereaved mothers, Janne and Rita (all names are pseudonyms), representing the peer organization, were recruited as consultants. During the process, they decided to develop and implement peer support for recently bereaved persons after drug-related deaths. They wanted to establish this service in collaboration with the crisis team in a medium-large town and formalize the work through a written cooperation agreement between the two organizations. The choice of this service intervention originated from Janne and Rita’s own experiences of not receiving help and support after the deaths of their children.

Janne contacted the special consultant of the family centre and requested a meeting with him in addition to relevant health and welfare services and their managers to establish and formalize the peer support initiative. Two meetings were held, and the managers were invited to both but did not attend (for an overview of the attendees of the meetings, see Supplementary Table I). In the first meeting, knowledge from the END project was presented as a rationale for why the intervention was important. The local crisis team became optimistic about collaborating with the peer organization, and the meeting agreed upon a need for a cooperation agreement between the two organizations. The peer support was implemented shortly after the first meeting, without any formal agreement between the stakeholders involved at that point.

The reason for the second meeting was to formalize the agreement. Because no representatives from the managerial level were present at the meeting, no formal agreement was reached. The attendees decided to bring the agreement draft to the decision-making level in the respective organizational lines (the peer organization and the public health and welfare service). However, due to unforeseen events and the absence of key persons, this did not happen. Discussions on how to reach a formal anchoring of the peer support service continued in the research circle meetings. A legal cooperation agreement was finally reached one year after establishing the service intervention. See Supplementary Table I for an overview of the meetings and participants in the first service intervention.

#### Service Intervention 2: A Monthly Low-Threshold Meeting Place for Bereaved Persons After Drug-Related Deaths

A sizable Norwegian public health and welfare service, represented by their interdisciplinary overdose coordinator, Trude, decided to establish a monthly meeting place for bereaved persons after drug-related deaths. The choice of service intervention was informed by Trude’s experiences of encountering bereaved persons requesting a place to meet with people in the same situation.

To facilitate the service intervention, Trude established a project group of seven participants who were jointly responsible for developing and implementing the meeting place. The members of the project group included employees from different organizational units within the public health and welfare service and the first author. The project group held frequent meetings during the development period (April—September 2022). The meetings aimed to clarify roles and responsibilities and to provide the innovators with sufficient knowledge to carry through the implementation process. The project group continued with regular meetings after the meeting place was established, and the service intervention was continuously adjusted and developed based on the attendees’ evaluations and advice, as well as ideas from the research circle. See Supplementary Table 2 for an overview of the meetings and participants in the second service intervention.

### Overview of the data material

Data from the development and implementation of the two new services were generated in the period February to November 2022 from the following sources: (a) minutes of meetings from the implementation process, (b) field notes from meetings and first author’s reflections, and (c) a transcript of audio recordings from research circle meetings.

### Research ethics

The Norwegian Regional Committees for Medical and Health Research Ethics (2017/2486/REK vest) and the Norwegian Agency for Shared Services in Education and Research (52551) have recommended the END project and the research circle study. All participants in the research circle were informed about the investigation and their rights as research participants (following the Declaration of Helsinki) and gave written consent. Employees participating in the second service intervention project group were informed about the first author’s role and the purpose of the research circle. They consented orally to participating in the research. Ethical dilemmas related to the vulnerable group to whom the service interventions were to be offered generated many reflective discussions in both the research circle and the project groups during the design and implementation period. The groups frequently discussed how to provide supportive services and avoid non-supportive elements in the service interventions. In the following presentation of results, all participants are given pseudonyms, and any other identifiable information has been redacted.

### Data analysis

The data generated were analysed using Braun and Clarke’s (2021) reflexive thematic analysis (RTA), consisting of six interconnected phases (1–6) that are well suited for multiple data sources. The data material was first read and re-read to gain an overview of its essence. Then, the data material from both service interventions was sorted chronologically, first separately and then together (1). The first and last authors (BM and MAR) coded the data material in separate Word documents (2). They extracted codes and initial themes based on the research questions separately and then together (3). All the authors participated in multiple re-extractions of the themes and consented to the presented themes (4–5). The first author started writing up the study (6) and finished it by collaborating with the others.

## Results

Two key dimensions were experienced as important for the implementation of the new services in the public health and welfare system when research circles were applied as a method for co-creation. The theme of “the inclusion of participants from different contexts” is presented first, followed by the second, “support structures for service interventions,” both with consecutive codes. In the sections below, (1) and (2) indicate the related service intervention.

### The inclusion of participants from different contexts

The results illustrated the importance of carefully considering who should be included in a research circle. The following section will describe how participants’ access to decision-making structures and resources were important dimensions for planning and progressing their interventions.

#### Access to decision-making structures

The participants’ organizational affiliations proved to play an essential role in their access to decision-making structures in the organizations where the new services were to be established. The research circle methodology implies that the service interventions created in research circles will be implemented outside the circles. Hence, there is no guarantee that the interventions will be met with responsiveness in the organizations where they are to be administered.

Janne and Rita represented a peer support organization. The results show they struggled to access the decision-making level in the organization they aimed to collaborate with:

My experience, then, is that you are, in a way, sitting with people at a slightly too low level for you to actually make any agreement. It’s difficult; they don’t have the authority to sign anything and have to take it back to their managers, and it might not be prioritized, and when the manager hasn’t been involved in the process, it might not be that important. (Rita (1), seventh research circle meeting)

The lack of allies in decision-making positions in the public health and welfare service became a barrier for Janne and Rita in formalizing their service intervention initiative. Correspondingly, Janne’s initial contact was made with a single person (a middle manager), who was the link to the public health service and became a pivotal person in gaining access to the authoritative structures in the organization. The results from the analysis illustrate how the formalization process was interrupted when the middle manager became absent for a period.

In the second service intervention, Trude, who represented a public organization, had a role immediately below the head of the department and a department-wide responsibility for service development. From this position, she provided information about the service intervention and its purpose, both upward and downward along the organizational lines, and she managed to anchor the service intervention to administrative units, as opposed to single individuals. The field notes from meetings in the project group and transcripts from research circle meetings consistently demonstrated how the anchoring of the service intervention progressed throughout the process. For example, in field notes from project group meetings (first and second meeting (2)), the participants discussed how employee rotation schedules should be changed and adapted so that the same staff could be present at all gatherings for the bereaved persons. The field notes from the seventh meeting (2) showed that the rotation schedule had changed, illustrating the power held by Trude and the project group. The opportunity to change organizational structures stands out as an essential difference between the two service interventions and probably contributed to a more rapid onset of the new services in the second service intervention.

#### Resources for service interventions

The research circle participants’ access to resources (staff, financial means, and location) necessary to establish new services was also an important dimension influencing the service intervention process and outcomes. Rita and Janne (1) had the resources to offer peer support. However, they needed supplementary resources to realize their service intervention, such as access to newly bereaved persons through the local crisis team, time allocated for the intervention work, and a place to provide peer support. A discussion frequently brought up in the research circle meetings was a fear that the public health and welfare service would not take up their part of the responsibility but instead hand it over to the peer organization. Rita reflects on whether the collaboration is real if it is not formalized or has joint responsibility:

There is something about the timing; it is very appropriate to say that you should collaborate with the volunteers, but the public sector will not take any responsibility for it. And when everything is left to the voluntary sector, and the public sector is in a way without obligation, then in a way, you have not entered a collaboration (…). If the public sector is completely exempted from everything in a way, then it’s just no longer an offer that is required by law that is important in a way; it becomes something that only the volunteers deal with. (Rita (1), eighth research circle meeting)

Rita was concerned with whether a service outcome/measure becomes less important if the responsibility is left to civil society alone. Following Rita’s statement above, there is a risk of devaluing the volunteers if the public sector is unwilling to collaborate with them.

The time allocated for the intervention work was also identified as a barrier during the co-creation process of the service interventions. In this regard, the structuring of the research circle as a collaborative network on top of the participants’ ordinary work evolved as an important factor. All participants in the present research circle were paid by their employer (or received a pension) for participation in research circle gatherings. Still, most participants were unpaid for related work between the meetings. The result showed that several participants in the research circle meetings communicated that insufficient time was allocated to design and implement the new services. The participants perceived the co-creation process as time-consuming for themselves and their organization (see Supplementary Tables I,2 for the immense size of the work). In the first service intervention, Janne and Rita had not set aside time for this in their ordinary work, as their activity with the peer organization was performed in their spare time. In a research circle meeting, Janne excused herself for not having time to work with the service intervention: “Yes, not much more than that has happened (…), but I have been very busy, as you know” (referring to other events in her personal life) (Janne (1), seventh research circle meeting).

In contrast, Trude (2) could work with the implementation of the new service as part of her regular work. Additionally, she could utilize other staff resources from her department, as illustrated by this quote: “I have received help; the local public health and welfare service pays 20% of the position for someone who will help me with the bereavement meeting place for the rest of the year” (Trude (2), eighth research circle meeting). Comparing the premises and access to resources for the two service interventions shows that access to or a lack of resources, including remunerated and allocated time, can be essential for the co-creation process and outcome. In addition, some mechanisms relating to civil society, such as the reliance upon volunteers, can be exploited by the public sector and inhibit the anchoring of long-lasting and binding structures.

### Support structures for service interventions

Another key dimension identified as important for the service interventions was the support structure provided by the research circle, which worked as a separate support unit parallel to the service interventions. The following section describes how the research circle contributed to creating a common frame of reference, with joint responsibility for helping each other to overcome barriers and improve the quality of the interventions. The first author’s role as a mediator between the participants and their organizations was also identified as essential for the process and outcomes of the service interventions.

#### Joint responsibility and commitment to intervention outcomes

The transcripts from the research circle illustrated that the predefined purpose of the research circle, to create and implement new services for bereaved persons after drug-related deaths, created a joint responsibility and engagement for the outcome of the interventions among the participants and contributed to the concentrated focus throughout the process. Furthermore, the transcripts showed that the participants generously shared reflections, resources, and advice from their network. Several research circle participants possessed a network that could be utilized as an additional resource to promote the progression of the service interventions. Frida aimed to help Janne and Rita (1) with their peer support intervention by mobilizing a person in her network who had the necessary resources to gain access to their organization:

Frida: I’m still hung up on her, the overdose coordinator in the health and welfare system (working where the peer support was to be implemented), because, as I said, I know Hanne very well. So, I say to you I’m going to help you by trying to contact Hanne.

Several participants: Yes, do it!

Frida: I have collaborated with Hanne for many years in relation to the national overdose strategy (…). I can call her within a week and hear how things are going in her public health service. (Frida and several participants, (1), eighth research circle meeting)

In the example above, Frida’s initiative led to a new key contact person, which came to play an essential role in Janne and Rita’s further efforts to maintain and re-establish contact with the public health and welfare service and the final fulfilment of a written cooperation agreement one year later.

The research circle meetings, as parallel structures to the service interventions, represented an arena for discussing barriers and challenges experienced by the participants. This possibility for joint discussions and reflections seemed to empower the research circle participants to become more confident that they had both research-based knowledge and experience from practice as a foundation for their interventions. Trude brought up some reflections with the research circle after arranging the first meeting with the bereaved individuals in her service intervention and asked for input from the research circle participants. Elin responded to this: “But, yes, you say, Trude, something about wanting to fix and fix things for them, but the best way to fix things for them is to give them good tools” (Elin, (2) ninth research circle meeting).

Furthermore, the field notes show how the participants transmitted the commitment from the research circle to their collaborative partners outside it. For example, in the first meeting of the project group, Trude emphasized the joint responsibility for creating the new services. In the following meetings, the field notes demonstrate that she invited the other participants in the project group to give feedback from the meeting place, asked for the project group’s reflections, and posed open questions about how to handle challenges that occurred. In the peer support service intervention, the engagement was “contagious,” and the local crisis team immediately became engaged after Janne and Rita’s presentation on the importance of peer support to persons recently bereaved after drug-related deaths.

#### The mediator role

Transcripts from the research circle meetings and the project group meetings showed that the first author’s role as an action researcher became important for facilitating the progress of the service interventions and mediating between the research circle and the two service interventions. As a mediator, the first author contributed with overviews of knowledge from the END project, giving research-based input to the content and design of the service interventions. The participants in the service interventions expressed in the research circle meetings that having the first author directly engaged together with them in the service intervention work was supportive, served as a legitimation of the need for new bereavement services, and increased the priority of bereaved persons after drug-related deaths in their organizations. In her organization, Trude described the first author’s role in the following terms: “as part of the group, in a way” (Trude (2), 10^th^ project group meeting).

In sum, the results from this study point to two key dimensions that proved important for the process and outcome of implementing new services through the research circle. While joint commitment and responsibility for the outcome stand out as important drivers in the co-creation process, the outcome still depends on participants’ ability to intervene in organizational structures. Notably, this highlights the importance of considering the composition of a research circle, particularly regarding the participants’ positions and roles, as this plays an important role the participants’ access to the decision-making structures and resources necessary for implementation. Another key dimension was the support structure made possible by the research circle as an ongoing parallel unit. Here, the participants could access ideas, knowledge, and networks to help them overcome implementation barriers and gain legitimacy for the intervention process. However, to fully utilize the potential inherent in the research circle as a method for implementing new services, it appeared important to have a person assigned a mediating role between the research circle and the context for implementation.

## Discussion

Based on the insights from the two service interventions described, we will discuss how research circles can be a method for co-creation and highlight some key dimensions important for the co-creation process and its outcome.

### Research circles as a method for co-creation and the increase of innovation capacity

This study shows that the research circle method provided three beneficiary aspects supporting co-creation possibilities. *Firstly*, providing an innovation unit separated from daily work promoted the realization of service interventions. It can be challenging to carry out innovative work alongside day-to-day operations, as the administrative tasks take time and mental capacity (Meyer et al., 2023). As such, the research circle served as what Meyer et al. (2023) describe as an innovation unit, which was physically separate from participants’ daily operational tasks. Designing one’s innovation units can contribute to reducing cultural and structural inertia, which can prevent organizational changes (Meyer et al., 2023).

*Secondly*, in parallel with the service intervention designs, the research circle meetings provided a space where the participants could triangulate information, questions, and reflections between the two units. The results show that this triangulation was essential in supporting the participants in their work with the interventions, adjusting the initiatives, and helping them manage barriers in the implementation process. The triangulation of information between the research circle participants contributed to strengthening the research circle participants’ capabilities, similar to what Meyer et al. (2023) describe as essential for building organizational innovation capacity through dynamic and complementary capabilities. The research circle participants emphasized dynamic capabilities such as sensing a challenge, seizing it, and contributing to transforming the organization to implement the new service, for example, by changing rotation schedules. In addition, complementary capacities such as physical premises and funding needed for the implementation process became subjects of investigations and discussions in the research circle. Through these processes, the participants became aware of what resources were required for the implementation and could provide input to the organization to arrange these capacities.

*Thirdly*, the continuous outward focus (and commitment) regarding how to intervene in the field of practice was important. Still, the participants had to work actively to avoid the service interventions remaining in the research circle, which is an identified challenge in research circle work (Persson, 2016). In this regard, the participants’ continuous work with the anchoring processes in the receiving organizations was important, such as, for example, getting in touch with the right decision-making level and gaining legitimacy to prioritize services for this population. Working in this way corresponds to Osborne’s (2021) view that co-creation occurs in an ecosystem with several levels. For the realization of new services and to increase public value, there is a need to see these levels as interrelated and work in the space between the levels to ensure connection.

### Key dimensions for the process and outcome – creating public value

The working process in this paper corroborates findings from other research circle studies that have found research circles to be a suitable framework for co-creation processes involving multiple stakeholders, including vulnerable citizens, in the public health and welfare system (Chalachanová et al., 2023; Follevåg & Seim, 2021; Follevåg et al., 2023; Löfqvist et al., 2019; Møgster et al., 2023; Witsø et al., 2022). Findings in these works are supported by a systematic review of knowledge that shows that co-creation generates positive outcomes in the form of public value for groups of vulnerable people if professionally facilitated (Acar et al., 2023). The examples presented above report outcomes related to more process-oriented aspects of research circle work, such as increased empowerment and participation among the research circle participants, which are also identified in this article. Empowerment and participation align with what Acar et al. (2023) frame as democratic outcomes of co-creation processes.

Additionally, an important key dimension for realizing the service interventions was the participants’ roles as engagers in relation to the organization and the support to manage this role. The research circle participants became key persons for transferring relevant ideas, information, and knowledge to the organizations where the new services would be implemented. In addition, they were important for creating engagement and motivation for change in the receiver organizations. Contributing to an organization’s collective engagement is essential for building organizational innovation capacity (Meyer et al., 2023). The role of bringing in new ideas and engaging the organization’s employees in implementing new services placed a lot of responsibility on the participants from the research circle. In this situation, the action researcher came to have an important role supporting the participants. The first author’s role as an action researcher can be seen as an intermediary, described by Haug (2023) as an actor contributing to services in the public sector who substantially differs in form from public service providers and service users. In this role, the first author became both a supporting resource and a legitimacy of the need for novel services by representing access to new knowledge, indicating a strong need for help and support services for bereaved persons after drug-related deaths. This contribution to increasing the quality of services for a vulnerable population can also be viewed as an outcome relating to Acar et al. (2023) democratic category of public value.

### The co-creation of public value through knowledge-based services

The service interventions were not derived directly from the new research but were intermingled with the input of bereaved persons. When working with the service interventions, the research circle functioned as a parallel support structure, allowing for merging the different knowledge types. The first author and other participants from the END project were able to provide the research-based knowledge, while the other participants supplied the experience-based and professional forms. This coheres with the idea that co-creation processes benefit from both lay actor and professional knowledge (Torfing et al., 2023). This triangulation of knowledge probably contributed to an increase in the quality of service interventions, and as such the creation of public value (Acar et al., 2023; Osborne, 2021).

As the bereaved mothers expanded their role from consultants to designers of their own service interventions, this might have led to the new interventions being more closely connected to the bereaved persons’ own needs. For example (Palumbo, 2016, p. 73), claims that co-creation involving users makes provisions “more consistent with their health-related needs.” The sharing of information and ideas that in the current work was made possible by the research circle methodology has also been confirmed in another study as important for expanding services, providing adequate ones, and contributing to service diversity and innovation in complex intervention situations (Yeo & Lee, 2020). Correspondingly, the ability to refer to new research-based knowledge seemed to increase the participants’ well-being and skills when implementing the new services and their likelihood of being perceived as legitimate by the receiving organization.

On the other hand, it is relevant to ask whether the aim of using research circles as a mean for implementing new research-based knowledge was fulfilled. The results show that both the two service interventions were designed based on a departure in the participants’ own experiences, and did not necessarily depart from what research pointed to as the most urgent need for service improvement. Questions can be asked regarding whether the two prevailing logics for service innovation in public sector, co-creation (Osborne, 2021; Torfing et al., 2019) and research-based innovations (Gulbrandsen et al., 2021), are compatible or contradictory. Including multiple stakeholders, partners, and networks in service interventions makes other knowledge relevant, including the experience-based type that can deviate from or even be in tension to the research-based form (Osborne et al., 2023). As such, the aim of reducing the research-practice gap and the delay in research-based implementation (Khan et al., 2021; Morris et al., 2011) can be challenged if not consciously addressed in innovation designs and the co-creation of public value outcomes. Osborne et al. (2023) suggest that these should be managed instead of overcome.

### Barriers to the co-creation of value between civil societies and the public sector

The key dimensions identified as important for the process and outcome also shed light on some barriers faced by the different participants when working with their service interventions. The choice of designing their own service interventions placed the bereaved mothers in a more complex collaborative situation than solely acting as consultants. The results show the bereaved mothers, in their role as co-initiators, struggled with gaining access to the decision-making structures in the collaborating public health and welfare service, an organizational barrier which, according to Cinar et al. (2019), is the most frequently identified obstacle in implementation processes.

Traditionally, in Nordic countries, the public system usually holds a co-initiator role, retaining formal power, responsibility, and control (Sørensen & Torfing, 2018). The change in who is holding the co-initiator role can explain why the two peer organization representatives experienced barriers regarding access to decision-making structures. Pestoff et al. (2006) has identified a tradeoff between the public and civil societies, in which the civil one is seldom adversarial or offers an alternative to governments but tends to be complementary or supplementary in function. The bereaved mothers’ change in role-taking could explain why it became labour-intensive for the bereaved mothers to come into position to implement their new peer-support service. In addition, the need for professional service providers to change their role from providing services to managing co-creation processes (Steen et al., 2018) may have influenced this result. The need for the role change of professionals coheres with Acar et al. (2023) relationship category of public value, as when this occurs, it will impact the working conditions of users participating in co-creation processes.

Additionally, the results indicate that involving service users as co-initiators requires changes in the current dominant discourses for public service production and innovations, an observation that adheres to Osborne et al. (2022) claim that public service organizations need to shift away from internal value chains towards external value creation.

## Conclusions and future research

The results from this study contribute to the growing body of research on outcomes in the public health and welfare sector. By employing the research circle as a co-creation method, the participants increased both their own and their organizations’ innovation capacity by providing ideas for new interventions and bringing in relevant knowledge and support. The participants were supported by the triangulation possibilities provided by the ongoing parallel meetings in the research circle and project groups, as well as the first author in an intermediary role. This structure proved to be important for maintaining progress in the service interventions. In this manner, research circles used as a co-creation method in the health and welfare sector can function as a separate innovative unit.

However, the study has identified some barriers to this type of co-creation. To overcome barriers to reaching decision-making levels in organizations where the new services are to be implemented, we suggest including stakeholders with access to decision-making levels in their organization in the research circle and, as such, creating better premises for the implementation of the research circle designs.

Due to scarce research on outcomes from research circles aimed at facilitating service interventions in the health and welfare sector, we suggest further studies are needed to evaluate the effects of new services designed and implemented through research circles. In addition, investigations are required to explore how barriers to co-creation can be better managed to facilitate external value creation. In this regard, we call for research examining the relationship between civil society and the public health and welfare system. Additional works on this topic can continue promoting conditions for creating integrative services rather than complementary ones.

## Supplementary Material

Supplementary tables revised.docx

Author Bio .docx
