# Supplementary material for: Research circles as a method for implementing new services in the public health and welfare system
Source: Int J Qual Stud Health Well-being. 2024 Jun 11;19(1):2366087. doi: 10.1080/17482631.2024.2366087 (PMC11172250; doi:10.1080/17482631.2024.2366087)
Supplement: Supplementary tables revised.docx [file ZQHW_A_2366087_SM4081.docx]

Supplementary Materials

**Research Circles as a Method for Implementing New Services in the Public Health and Welfare System**

| **Date** | **Type of meeting** | **Participants** | **Topic** |
| --- | --- | --- | --- |
| February  02.15.22 | First meeting | Janne, Rita, the first author, and four public servants, including a special consultant from the family center | Investigating public health and welfare service situation and the need for knowledge and support from the peer organization. Presenting END project knowledge and offering peer support |
| March  3.29-30.22 | Sixth research circle meeting | Janne, Rita, the first author, and research circle members | Information about the service intervention, cooperation agreement draft, and start-up |
| April  04.06.22 (Teams) | Planning meeting | Janne, Rita, and the first author | The agenda for the next meeting with the public health and welfare system: how to design the cooperation agreement draft design |
| April  04.19.22 (Teams) | Planning meeting | Janne, Rita, and the first author | Designing a cooperation agreement draft |
| April  04.26.22 | Second meeting | Janne, Rita, the first author, and three public servants, including a special consultant from the family center | Presenting cooperation agreement draft and revisions |
| June  06.07.22 | Seventh research circle meeting | Janne, Rita, the first author, and research circle members | Unclear roles in the public health and welfare system |
| September 09.12.22 | Eighth research circle meeting | Janne, Rita, the first author, and research circle members | Cooperation agreement draft and how to move forward when the special consultant from the family center is absent |
| October 10.18.22 | Ninth research circle meeting | Janne, Rita, the first author, and research circle members | Identifying new contact persons. Continuing to reestablish contact |
| November 11.28-29.22 | 10^th^ research circle meeting | Janne, Rita, the first author, and research circle members | Evaluation and knowledge sharing |

**Supplementary Table 1.** Overview of meetings in the development and implementation period of new service 1 (service intervention 1): peer support for recently bereaved persons after drug-related deaths. Research circle meetings are marked with gray.

| **Date** | **Type of meeting** | **Participants** | **Topic** |
| --- | --- | --- | --- |
| March  03.29-30.22 | Sixth research circle meeting | The project leader and first author | Decision on what to do and how to start |
| April  04.29.22 | First project group meeting | The project leader, first author, and project group | Clarification of the project group’s needs: END project knowledge and getting to know each other |
| May  05.12.22 | Second project group meeting | The project leader, first author, and project group | Presentation of END project knowledge: help needs and experiences for bereaved persons after drug-related deaths. Discussion about how to design the meeting place |
| May (Teams)  05.20.22 | Third project group meeting | The project leader, first author, and project group | How to create a community for bereaved persons. Summing up further needs for resources |
| May  05.23.22 | Fourth project meeting | The project leader, first author, and project group | Presentation of END project knowledge: stigma. How to reduce stigma through the use of non-stigmatizing language |
| June  06.07.22 | Seventh research circle meeting | The project leader and first author | How to promote the low-threshold meeting place, use of language in a non-stigmatized form in information material |
| June  06.08.22 | Fifth project group meeting | The project leader, first author, and project group | Feedback from the research circle meeting promoting the low-threshold café. How to organize resources. Offer knowledge and information sessions for the bereaved persons in gatherings |
| June  06.10.22 | Full-day course by a regional competence center | The project leader, first author, and project group | Crisis intervention, the special grief involved in bereavement after drug-related deaths |
| June  06.23.23 (Teams) | Sixth project group meeting | The project leader and project group | Information about, need for, and provision of resources |
| August  08.11.22 | Seventh project group meeting | The project leader, first author and project group | Risk assessments, including those of bereaved persons and attendees, and the need for extended help. Information materials |
| August  08.23.23 | Eighth project group meeting | The project leader, first author, and project group | Physical meeting locations to be used and review of routines for use |
| September  09.07.23 | First low-threshold meeting | 11 bereaved persons, as well as  the project leader, the first author, and four from the project group | Theme: what is a low-threshold meeting place, and how do you (the bereaved persons) want it to be designed? Conversations in several smaller groups |
| September  09.12.23 | Eighth research circle | The project leader and first author | Evaluation of the first low-threshold meeting: how to include all bereaved persons, start a group formation, e.g., through common topics such as grief |
| September  09.20.23 (Teams) | Ninth project group meeting | The project leader, first author, and project group | Evaluation of the first low-threshold meeting. How to start a group formation and include all bereaved persons, e.g., through common topics such as grief |
| October  10.05.22 | Second low-threshold meeting | Eight bereaved persons, as well as  the project leader, the first author, and five from the project group | The END project and knowledge (after request from the bereaved persons). Conversations in a larger group and one smaller group |
| October  10.18.22 (Teams) | Ninth research circle meeting | The project leader and first author | Evaluation of the second low-threshold meeting |
| October  10.19.22 (Teams) | 10th project group meeting | The project leader, first author, and project group | Evaluation of second low-threshold meeting. Adjustments to staff needs (reduce after a start-up period) |
| November  11.02.22 | Third low-threshold meeting | Eight bereaved persons,  four from the project group | The services available in the public health and welfare system: rapid mental health care, follow-up services, and local crisis team |
| November  11.28-29.22 | 10^th^ research circle meeting | The project leader and first author | Evaluations so far and knowledge establishment sharing |

**Supplementary Table 2**. Overview of meetings in the development and implementation period of the new service 2 (service intervention 2): a monthly low-threshold meeting place for bereaved persons after drug-related deaths. Research circle meetings are marked with gray.
