# Supplementary material for: Research circles as a method for implementing new services in the public health and welfare system
Source: Int J Qual Stud Health Well-being. 2024 Jun 11;19(1):2366087. doi: 10.1080/17482631.2024.2366087 (PMC11172250; doi:10.1080/17482631.2024.2366087)
Supplement: Author Bio .docx [file ZQHW_A_2366087_SM4080.docx]

**Author Biography**

**Birthe Møgster** is a doctoral research fellow in a national project on Drug-death Related Bereavement (the END project) at the Department of Welfare and Participation, Western Norway University of Applied Sciences. She is educated as social educator with a master’s degree in administration and leadership in health and social welfare systems. Her research interests include research circles, bereavement research, public management, and health and social services innovation. She has a long-lasting clinical practice as a leader and milieu therapist in substance use services.

**Ottar Ness, PhD,** works as Professor at Norwegian University of Science and Technology (NTNU) and Head of NTNU WellFare: Nordic Research Center for Well-being and Social Sustainability. His research interests are in Recovery and Mental Health, Citizenship, Mattering and innovation in the public sector. He is Adjunct Professor at University of Agder (Norway) and Aalborg University (Denmark).

**Monika Alvestad Reime** is post doctor at the Department of Welfare and Participation,  Western Norway University of Applied Sciences. She is educated as social worker and has a PhD in Administration and Organization theory from the University of Bergen. Her research interests include public policy, child welfare, grief and bereavement research, and innovation in health and social services. She is now working full-time in a national project on Drug-death Related Bereavement and Recovery (the END-project).
